# Adolescent suicidal ideation: dissecting the role of sex in depression and NSSI predictors

**DOI:** 10.1186/s13034-024-00741-z

**Published:** 2024-06-06

**Authors:** Zi-Ye Huang, Qian-Nan Ruan, Yawen Zheng, Heng Miao, Yu-Wei Wu, Wen-Jing Yan

**Affiliations:** 1 Wenzhou Seventh People’s Hospital, Wenzhou, China; 2 School of Mental Health, Wenzhou Medical University, Wenzhou, China; 3 Lishui Second People’s Hospital, Wenzhou Medical University, Lishui, China; 4 Student Affairs Division, Wenzhou Business College, Wenzhou, China; 5 Zhejiang Provincial Clinical Research Centre for Mental Illness, Affiliated Kangning Hospital, Wenzhou Medical University, Wenzhou, China; 6 Wenzhou Key Laboratory of Basic and Translational Research for Mental Disorders, Wenzhou Medical University, Wenzhou, China

**Keywords:** Suicidal ideation, Depression, Non-suicidal self-injury, Adolescents, Sex differences, Network analysis, Logistic regression

## Abstract

**Background:**

Suicidal ideation (SI) is increasingly prevalent among adolescents, often arising from depression and linked with non-suicidal self-injury (NSSI). Previous studies have noted significant sex differences in the manifestation and predictors of SI, depression, and NSSI.

**Aim:**

This study aims to analyze and compare the relationships between SI, depression, and NSSI among male and female adolescents, examining whether these associations differ based on sex.

**Methods:**

A total of 368 adolescents (*M* = 15.43, *SD* = 1.22, about 56.2% female participants), both from clinical and school settings, were assessed for SI, depression, NSSI, and other related variables. Network analysis was utilized to explore the interconnections among these variables, focusing on identifying sex-specific patterns. Logistic regression was used to confirm the findings from the network analysis.

**Results:**

The network analysis revealed significant sex differences in the relationships between SI, depression, and NSSI. In the female network, the edge weights between SI and NSSI (0.93) and between SI and depression (0.31) were much higher compared to the male network (0.29 and 0, respectively). Centrality indices (strength, betweenness, closeness, and expected influence) for SI, NSSI, and depression were also higher in the female network. Logistic regression confirmed these findings, with depression being a potential predictor of SI only in females (OR = 1.349, *p* = 0.001) and NSSI having a stronger influence on SI in females (OR = 13.673, *p* < 0.001) than in males (OR = 2.752, *p* = 0.037).

**Conclusion:**

The findings underscore the necessity of considering sex differences when predicting suicidal ideation from depression and NSSI in adolescents. Intervention and prevention strategies should be tailored to address these distinct patterns in male and female adolescents.

**Supplementary Information:**

The online version contains supplementary material available at 10.1186/s13034-024-00741-z.

## Introduction

Suicidal ideation (SI), defined as thinking about, considering, or planning suicide, ranges from wishing one were dead to having a specific plan to end one’s life. SI is exhibited by almost all attempters [1] and can be categorized as either active or passive. Active SI involves a clear desire and intent to die, while passive SI refers to a desire to die without taking action [2]. The peak onset of SI occurs during adolescence, with prevalence ranging from less than 1% at age 10 to 17% by age 18 [3]. In China, suicide is the fifth leading cause of death and the primary cause of death among young adults aged 15 to 34 [4]. A cross-sectional study of 12,733 Chinese children and adolescents aged 9 to 18 revealed a 32% prevalence of SI [5]. This high prevalence of SI during adolescence may be attributed to the imbalance between cognitive capacity and immaturity in cognitive control, self-referential processing, and emotional reactivity [6].

Depression and non-suicidal self-injury (NSSI) have been identified as key factors of SI among adolescents [7]. Depression is a prominent factor in SI among youths [8] and is frequently diagnosed among adolescents who attempt suicide [9]. Studies have found moderate correlations, ranging from 0.40 to 0.60, between depression and SI [10]. In a study of 877 teens, symptoms of depression as early as Grade 9 accurately predicted SI in early adulthood, with females suffering from depression at much higher rates than males [11]. NSSI, an important risk factor for concomitant or subsequent suicidal behavior or ideation [9], has been found to be a predictive factor of elevated levels of SI and suicide attempts over a 2.5-year interval in a diverse adolescent sample [12]. Recent studies have further highlighted the relationship between NSSI and SI, with NSSI age of onset being a risk factor for NSSI severity and suicidal behavior [13, 14] and NSSI serving as a gateway to suicide in young adults [15].

Sex differences have been observed in SI, depression, and NSSI. Female adolescents are more likely to have suicidal thoughts, ideation, and depressive symptoms compared to their male counterparts [16, 17]. The sex ratio (women: men) of depressive disorders is greater than 1.7 for lifetime prevalence and 1.4 for 12-month prevalence after the age of 18 ^17^. This sex difference in depression rates first emerges in adolescence and continues into old age [18], although the gap is less pronounced in adults [19]. Regarding NSSI, the literature suggests that boys and girls engage equally in NSSI in non-clinical samples, although specific characteristics of the behavior may differ between sexes [20]. In clinical samples, girls tend to engage more frequently in NSSI [21].

The risk relationship between SI and depressive symptomatology also varies by sex. One study found that females had a higher risk of SI at moderate levels of depression compared to males, contributing to the overall higher level of ideation [22]. However, another study showed that SI was higher among male participants, with age being the predictor of SI for males and depression and loss of motivation (components of hopelessness) being the predictors for females [23].

Recent research has emphasized the importance of examining concurrent changes in NSSI and SI. Muehlenkamp et al. [24] found that changes in NSSI frequency were positively associated with changes in SI and suicide attempts, highlighting the dynamic relationship between these behaviors. Despite the growing body of literature on the associations between SI, depression, and NSSI, there is a need for a more comprehensive understanding of the complex interplay among these factors and how they differ by sex.

Network analysis provides a novel approach to understanding the intricate relationships among SI and related factors. By considering SI as part of a network, this method offers a more comprehensive understanding of SI itself and helps explore sex differences in the relationships between SI and related factors. A tightly connected network with strong connections among symptoms is considered risky, as the activation of one symptom can quickly spread to others, leading to more chronic symptoms over time [25]. Moreover, network analysis employs regularization methods (e.g., LASSO regression, ridge regression) to prevent overfitting and select the most effective parts among multiple factors, resulting in clearer and more realistic relationships that better reflect sex differences in the associations between SI and other factors.

The present study aims to utilize network analysis to describe sex differences in the relationship between SI and other factors, with a focus on depression and NSSI. By promoting a deeper understanding of the mechanisms underlying SI, particularly the relationships among depression, NSSI, and SI, this study seeks to provide insights into ways of improving clinical interventions. Given the sex differences in the factors influencing SI, it is crucial to tailor interventions to the individual, as a one-size-fits-all approach may render the intervention less effective.

Based on the existing literature, we hypothesize that:

1. The network structure of SI and related factors will differ between males and females.
2. Depression and NSSI will have stronger associations with SI in females compared to males.
3. The risk relationship between SI and depressive symptomatology will be more pronounced in females than in males.

By addressing these hypotheses, the present study aims to contribute to the growing body of research on sex differences in SI and its predictors, ultimately informing the development of more targeted and effective interventions for adolescents at risk of suicidal ideation and behavior.

## Methods

### Participants

The sample consisted of 483 adolescents aged 12 to 17 years (M = 15.52, SD = 1.305; 58% female) from a psychiatric hospital and two secondary schools in China. The hospital sample included 216 outpatients who had one-on-one mental health assessments conducted by psychiatrists. The school sample consisted of 267 randomly selected middle school students who completed self-rating assessments while being monitored by their teachers in their respective classrooms (i.e., group testing).

Inclusion criteria were adolescents aged 12 to 17 years, capable of understanding and completing the assessments, and who consented (from their guardians) to participate. Exclusion criteria included severe cognitive impairments that might interfere with understanding or completing the assessments, and failure to pass the manipulation checks, designed to assess sincerity and attention to the questionnaire’s instructions.

### Procedure

For the hospital sample, patients were briefly introduced to the purpose of our study and asked to complete the relevant scales based on the previous week, after entering the psychological assessment room. They could ask their psychiatrist for help if they had any questions. Prior to the mental health assessment, the patient and parent/guardian were informed about the assessment process and the scales to be used, followed by the signing of an informed consent form by the parent/guardian and the patient, as required by local hospital ethics regulations. When the task was completed, the psychiatrist checked to make sure that all portions were completed, and the subject was asked to leave the assessment room.

The school sample conducted their self-rating assessments while being monitored by their teachers in their respective classrooms (i.e., group testing). All participants signed an informed consent form and were explained the rules regarding anonymity, confidentiality, and their right to quit the experiment.

The respondents from the hospital were all included because the psychiatrists ensured carefulness in the one-on-one tests. However, considering that some students from schools may not have taken the group test seriously, we used a manipulation check in their questionnaires [26]. Students were asked to select D for “This question has other uses, please select D.” If students did not select D, they were excluded from the data analysis. We included two similar questions serving as checks; those failing one of the items were excluded. Of the total 267 respondents, 115 were excluded because they failed the manipulation check. Therefore, a total of 368 participants (M = 15.43, SD = 1.22; 60% female) were included in the data analysis.

The authors assert that all procedures contributing to this work comply with the ethical standards of the relevant national and institutional committees on human experimentation and with the Helsinki Declaration of 1975, as revised in 2008. All procedures involving human subjects/patients were approved by IRB in Wenzhou Seventh People’s Hospital (EC-KY-2,022,048).

### Measures

#### Self-injurious thoughts and behaviors interview

We used the Self-Injurious Thoughts and Behaviors Interview (SITBI) to assess adolescents in the clinical setting. The SITBI is a structured interview that assesses the presence, frequency, and characteristics of a wide range of self-injurious thoughts and behaviors, including SI, suicide plans, gestures of suicide, suicide attempts, and NSSI [27]. A previous study [28] reported a Cronbach’s α of 0.77 for the Chinese version of the SITBI in a sample of adolescents, demonstrating good internal consistency reliability. To merge the data for analysis in the present study, both groups of the participants answered yes or no to questions “Have you ever had thoughts of killing yourself?” regarding SI and “Have you ever had thoughts of purposely hurting yourself without wanting to die? (for example, cutting or burning)” regarding NSSI. Note that these are about their lifetime prevalence of NSSI and SI.

#### Hospital anxiety and depression scale

The Hospital Anxiety and Depression Scale (HADS) assesses both anxiety and depression, which commonly coexist [29]. The measure contains a total of 14 items, including seven for depressive symptoms (HADS-D) and seven for anxiety symptoms (HADS-A), with a focus on non-physical symptoms. An example item from the HADS-D is “I still enjoy the things I used to enjoy,” rated on a 4-point Likert scale (0 = definitely as much, 3 = hardly at all). Higher scores indicate more severe symptoms. One previous study [30] reported Cronbach’s α of 0.76 and 0.79 for the anxiety and depression subscales in Chinese adolescents, and confirmed the two-factor structure of the HADS through confirmatory factor analysis, supporting its construct validity.

#### Difficulties in emotion regulation scale

The Difficulties in Emotion Regulation Scale (DERS) is a 36-item self-report measure of six facets of ER. Items are rated on a scale of 1 (“almost never” [0–10%]) to 5 (“almost always” [91–100%]) [31]. Higher scores indicate more difficulty with ER. The psychometric properties of the DERS and its subscales are described herein. The Cronbach’s alpha values of the scale and six subscales range from 0.88 to 0.96 and test-retest reliability from 0.52 to 0.77. The results of the measurement conform to the necessary psychometric properties. It is suitable for measuring the degree of difficulty with emotion regulation in Chinese adolescents [32].

#### SPSI-R

There have been several revised versions of the SPSI-R for use in the Chinese language [33]. The present study used the Chinese version, which shows both good reliability and validity. The scale consists of 52 items rated on a 5-point Likert scale (0 = not at all true of me, 4 = extremely true of me). An example item is “When my first efforts to solve a problem fail, I become uneasy about my ability to handle the situation.” The overall Cronbach’s alpha is 0.85, and the rational problem-solving (RPS), avoidance style (AS), negative problem orientation (NPO), positive problem orientation (PPO), and impulsivity/carelessness style (ICS) subscale values are 0.85, 0.82, 0.70, 0.66, and 0.69, respectively. The SPSI-R uses a five-point Likert-type scale ranging from 0 to 4.

### Data analysis

**Fig. 1:**
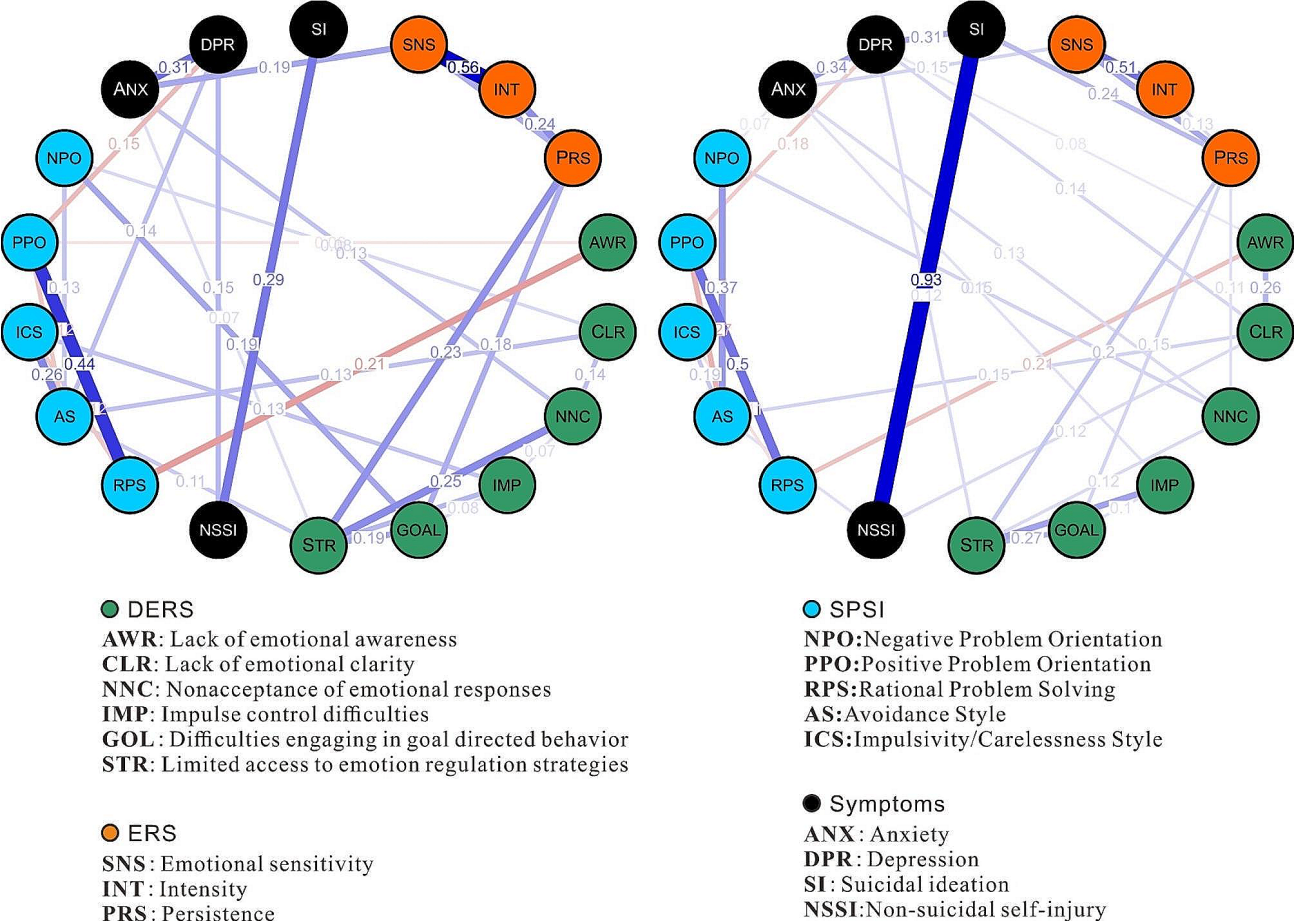
Two estimated network structures based on 160 males (left) and 205 females (right). The networks show the relationships among variables, including symptoms (SI, NSSI, depression, and anxiety) and psychological characteristics (ERS, SPSI, and DERS). The edge weights are the regression coefficients with regularization. Blue edges represent positive relationships and red edges indicate negative relationships. The thickness of the edge reflects the magnitude of the relationship

Network analyses with frequency variables of NSSI and SI were conducted in R using the mgm package, which allows for the explicit estimation of graphical models using mixed variable types [34]. The parameters were specified as lambdaGam = 0.25 and alphaGam = 0.25 in the mgm function. The glmnet package was used to provide regression analysis with L1 and/or L2 regularization. With this algorithm, one variable was taken as Y and the others as predictors, resulting in a coefficient matrix illustrating the relationships among these variables. Based on this coefficient matrix, the network could be plotted (see Fig. 1).

**Fig. 2:**
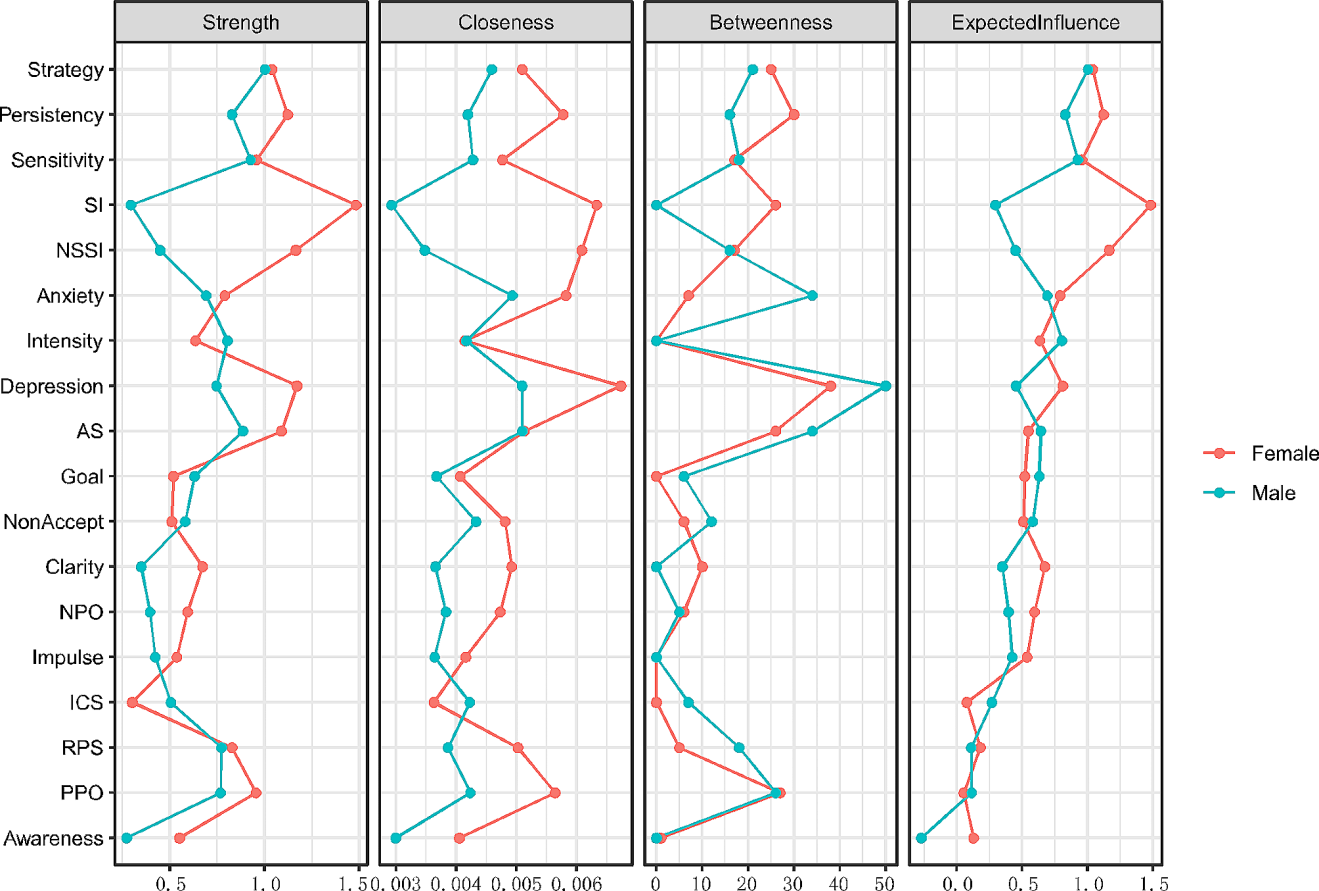
Centrality indices for the nodes in the male and female networks, including those for strength, betweenness, closeness, and expected influence. Red and blue represent female and male networks, respectively

We calculated several indices of node centrality to identify the symptoms or components most central to the network. For each node in the male and female networks, we calculated the strength (i.e., the absolute sum of edge weights connected to a node), closeness (i.e., the average distance from the node to all other nodes in the network), betweenness (i.e., the number of times a node lies on the shortest path between two other nodes), and expected influence (i.e., the sum of edge weights connected to a node) (see Fig. 2).

Additionally, we conducted logistic regression analyses to examine the relationships between NSSI, SI, and their related factors (i.e., depression, anxiety, emotion regulation difficulties, and problem-solving abilities) separately for males and females. The dependent variables were the presence of SI (0 = no, 1 = yes), while the independent variables were the scores on the NSSI, HADS-D, HADS-A, DERS, and SPSI-R subscales.

## Results

The prevalence of NSSI was 55.6% for females and 36.3% for males, while the prevalence of SI was 59.5% for females and 43.1% for males. There were significant differences between sexes across various variables (see Table 1). Females had significantly higher scores than males for SI (59.5% vs. 43.1%), NSSI (55.6% vs. 36.3%), depression (t = 1.40, *p* < 0.01), anxiety (t = 2.12, *p* < 0.001), ERS (t = 3.97, *p* < 0.001), and DERS (t = 5.12, *p* < 0.001). Note that these are about their lifetime prevalence of NSSI and SI.

**Table 1:** Descriptive statistics for the measurements

			Suicidal ideation		NSSI		Depression		Anxiety		ERS		DERS	
		*N*	*N*	%	*N*	%	*M*	*SD*	*M*	*SD*	*M*	*SD*	*M*	*SD*
Sex	Female	205	122	59.5	114	55.6	8.17	5.00	9.21	4.79	48.96	20.73	109.58	29.11
	Male	160	69	43.1	58	36.3	6.76	4.67	7.09	4.92	40.18	21.27	94.60	25.80
Source	Clinical	206	122	59.2	107	51.9	8.30	5.39	8.76	5.36	46.64	22.10	106.65	30.41
	School	159	69	43.4	65	40.9	6.58	4.00	7.66	4.31	43.13	20.33	98.37	25.57
Family structure	Regular	271	115	42.4	108	39.9	6.56	4.57	7.41	4.59	41.97	21.11	98.17	27.40
	Single parent	36	23	63.9	18	50.0	9.03	4.44	9.86	5.61	51.14	20.94	110.39	27.06
	Reconstituted	17	17	100.0	14	82.4	10.53	4.32	10.82	4.94	52.47	19.05	110.29	30.14
	Orphan	1	1	100.0	1	100.0	12.00		12.00		67.00		152.00	
Economic status	Rich	3	0	0.0	0	0.0	2.33	1.53	4.67	4.73	29.33	22.74	78.67	17.56
	Wealthy	86	35	40.7	37	43.0	6.05	4.56	6.62	4.40	38.58	21.81	94.51	26.95
	Normal	224	114	50.9	98	43.8	7.42	4.61	8.38	4.90	45.49	20.67	102.83	28.13
	Poor	11	6	54.5	5	45.5	7.91	5.24	7.45	4.18	45.27	21.18	98.64	26.18

In the networks, for males, approximately 31 of all 153 (80%) network edges were set to zero by the regularization; this value was 33 (78.4%) for females. Figure 1 shows the network for the variables, including symptoms (SI, NSSI, depression, and anxiety) and psychological characteristics (ERS, SPSI, and DERS). The values on the edges show the weights between the nodes; thicker edges mean stronger relationships.

The difference in network structure between the two sexes was very clear. The weights for the SI and NSSI were 0.29 and 0.93 for the male and female network graphs, respectively, implying that the association between SI and NSSI was significantly greater for female adolescents than for males. The weight between SI and depression in the female network graph was 0.31, while it was reduced to 0 for the male network graph, implying that SI in males is not directly related to depression or is much weaker than the relationship is for females. The weights for the SI and emotion persistence from ERS were 0 and 0.24 for the male and female network graphs.

Figure 2 shows the centrality indices. SI had a much stronger connection to other nodes in the female than in the male network across four centrality indices: strength, betweenness, closeness, and expected influence. In the female network, NSSI and depression also showed higher values for strength, closeness, and expected influence. As for betweenness, *depression* ranked the highest in both the male and female networks, as indicated by the shortest path between two other nodes. For closeness, *depression* ranked the highest only in the female network, meaning that it was closest to all other nodes.

### Verification of the results by logistic regression

Network analysis provides a global description for the relationships among the variables but doesn’t provide inferential statistics. Therefore, we used logistic regression, a classical approach, to verify the findings concerning sex differences when using depression and NSSI to predict SI among adolescents.

**Fig. 3:**
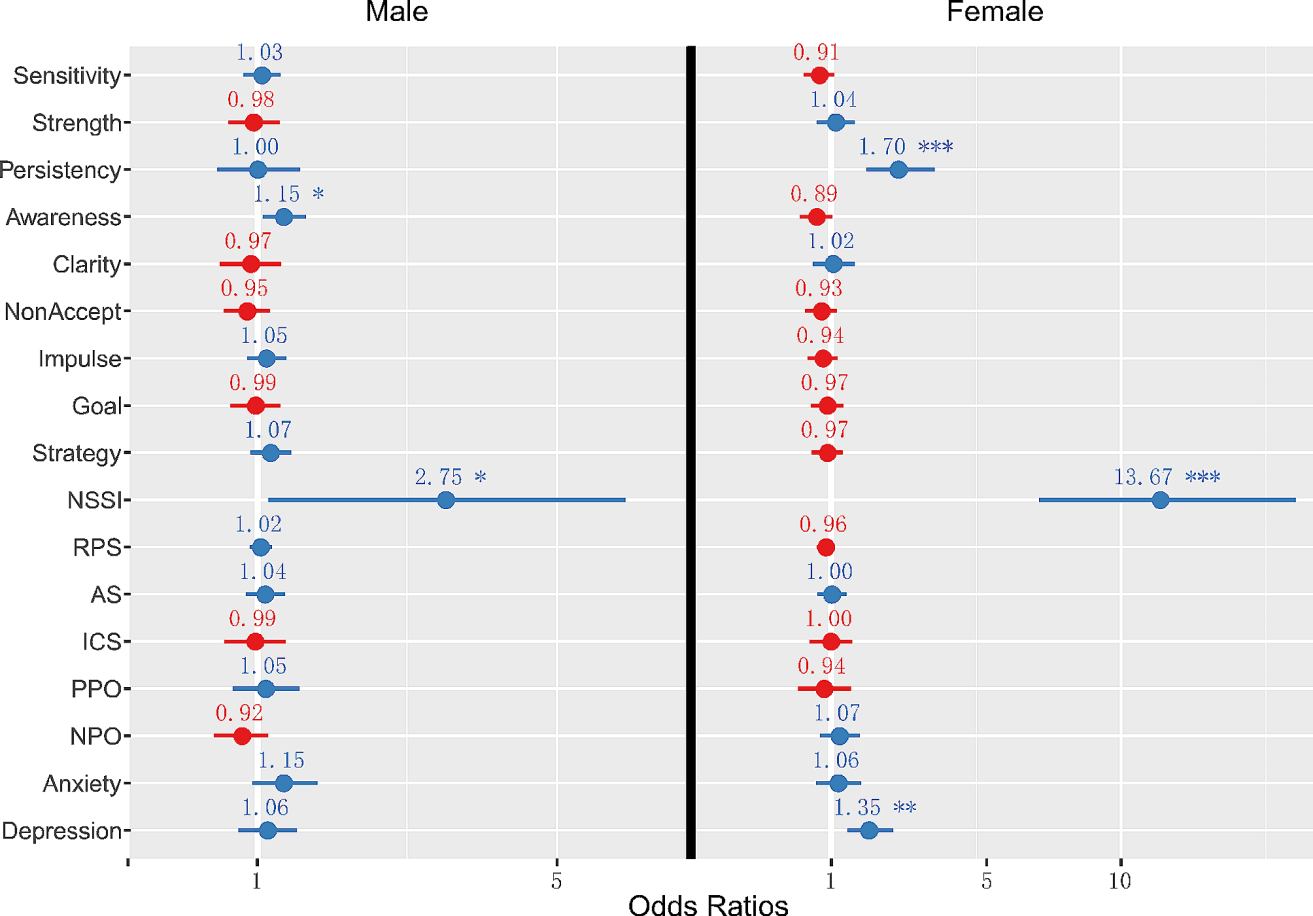
Forest plot of the odds ratios in the logistic regression. The red numbers indicate ORs less than 1 and the blue numbers indicate ORs equal to or greater than 1. Note that the OR value is large for NSSI because it’s a categorical variable

When all the samples were included, NSSI, depression, and emotion persistency were potential predictors of SI (for more details, see the Additional file materials 1). Here, our focus was on sex differences. The regression coefficient for depression was 0.056 for males (*p* = 0.482, *OR* = 1.058) and 0.299 for females (*p* = 0.001, *OR* = 1.349), indicating that depression is a predictor of SI only among females. For NSSI, the regression coefficient was 1.012 (*p* = 0.037, *OR* = 2.752) for males and 2.615 (*p* < 0.001, *OR* = 13.673) for females, indicating that SI is more highly influenced by NSSI in females. As for ER and emotional reaction characteristics, a lack of emotional awareness was a potential predictor in males (*B* = 0.142, *p* = 0.015, *OR* = 1.153) but not in females (*B* = -0.117, *p* = 0.077, *OR* = 0.890); emotion persistency was a potential predictor for females (*B* = 0.533, *p* < 0.001, *OR* = 1.705) but not for males (*B* = 0.003, *p* = 0.976, *OR* = 1.003). See Table 2; Fig. 3 for details. Therefore, the logistic regression analysis showed that depression and NSSI had a much more positive influence on females than males; emotional awareness was a potential predictor only for males, while emotion persistency was a potential predictor only for females.

**Table 2:** Logistic regression performed to ascertain the effects of various factors on the likelihood of SI

	Male				Female			
	*B*	*SE*	*p*	*OR*	*B*	*SE*	*p*	*OR*
Sensitivity	0.026	0.051	0.604	1.027	− 0.095	0.062	0.122	0.909
Intensity	− 0.019	0.071	0.791	0.981	0.035	0.078	0.653	1.036
Persistency	0.003	0.112	0.976	1.003	0.533	0.138	0.000	1.705
Lack of awareness	0.142	0.058	0.015	1.153	− 0.117	0.066	0.077	0.890
Clarity	− 0.034	0.083	0.681	0.966	0.015	0.085	0.857	1.015
Nonacceptance	− 0.054	0.063	0.396	0.948	− 0.078	0.065	0.229	0.925
Impulses	0.050	0.053	0.344	1.051	− 0.067	0.061	0.274	0.935
Goals	− 0.008	0.069	0.906	0.992	− 0.032	0.066	0.627	0.969
Strategies	0.072	0.055	0.195	1.074	− 0.032	0.063	0.617	0.969
RPS	0.019	0.031	0.532	1.019	− 0.045	0.036	0.214	0.956
AS	0.043	0.053	0.413	1.044	0.003	0.059	0.958	1.003
ICS	− 0.011	0.084	0.899	0.989	− 0.004	0.086	0.967	0.996
PPO	0.046	0.091	0.616	1.047	− 0.058	0.107	0.590	0.944
NPO	− 0.081	0.073	0.269	0.922	0.064	0.081	0.428	1.067
Anxiety	0.142	0.088	0.107	1.153	0.055	0.091	0.548	1.056
Depression	0.056	0.080	0.482	1.058	0.299	0.092	0.001	1.349
NSSI	1.012	0.486	0.037	2.752	2.615	0.517	0.000	13.673

## Discussion

The present study investigated networks of sex differences in the prediction of SI. We explored the connections among symptoms (SI, NSSI, depression, and anxiety) and psychological characteristics (ERS, SPSI, and DERS). The results showed that depression is positively related to SI only in females and not in males. NSSI is a much stronger related factor for SI among females than males. A lack of emotional awareness is a potential related factor only for males, while emotion persistency is a related factor only for females.

The stronger association between SI and NSSI in female adolescents is consistent with the interpersonal theory of suicide, which posits that individuals who engage in NSSI may develop an acquired capability for suicide through habituation to pain and fear of self-injury [35]. Our findings suggest that this process may be more pronounced in female adolescents, potentially due to their higher rates of NSSI [21] and greater sensitivity to interpersonal stressors [36]. This sex difference in the relationship between NSSI and SI highlights the need for sex-specific risk assessment and intervention strategies.

The current study also supports the notion that depression is a prominent factor in SI among youths [8], particularly in female adolescents. This finding aligns with the sex paradox of suicidal behavior, which refers to the phenomenon whereby females have higher rates of suicidal ideation and attempts, while males have higher rates of completed suicide [37]. Recent research [38] has attributed this paradox to the higher prevalence of depression and internalizing disorders among females, as well as sex differences in coping strategies and help-seeking behaviors.

Our findings suggest that lack of emotional awareness is a potential predictor of SI in males, while emotion persistence is a related factor of SI in females. These results align with the emotion dysregulation model of psychopathology [39], which posits that difficulties in regulating emotions can contribute to the development of various mental health problems, including SI. However, our study extends this model by revealing sex-specific patterns in the relationship between emotion regulation, emotion reaction, and SI. These differences may be rooted in socialization processes and gender norms that shape the development of emotional awareness and persistence differently in males and females [40]. For instance, males may be less encouraged to attend to and express their emotions, leading to a lack of emotional awareness that increases SI risk. In contrast, females may be more prone to rumination and prolonged emotional reactions, which could exacerbate SI risk through emotion persistence. Understanding these sex differences in emotion regulation and reaction has important clinical implications, as it suggests the need for targeted interventions that enhance emotional awareness in males and reduce maladaptive emotion persistence in females to prevent and treat SI effectively.

While our findings generally align with the existing literature, some contradictions were observed. For instance, Ibrahim et al. [23] found that SI was higher among male participants, which contrasts with our results indicating a higher prevalence of SI in female adolescents. This discrepancy may be attributed to differences in sample characteristics, cultural contexts, or the specific related factor examined in each study. It is possible that the relationship between gender and SI may be moderated by cultural factors, such as gender role expectations and socialization processes [37], which vary across different societies and may influence the expression and reporting of suicidal ideation.

Additionally, our findings suggest that depression is a related factor of SI in female adolescents, but not in males, which differs from some previous studies that have found depression to be a significant related factor of SI in both sexes [10, 11]. These contradictions may be explained by methodological differences, such as the use of different measures of depression and SI, or variations in sample characteristics, such as age range and clinical status. It is also possible that the relationship between depression and SI may be moderated by other factors, such as coping styles, social support, and life stressors [41], which may vary across different studies and populations.

These contradictions highlight the need for further research to clarify the complex relationships between SI, depression, and sex differences, taking into account potential moderating factors and using standardized measures and diverse samples. The inconsistencies in the literature also underscore the importance of considering the theoretical frameworks that guide the interpretation of findings, as different theories may provide alternative explanations for the observed sex differences in the related factors of SI.

### Limitations

First, the reliance on self-report measures may have introduced response bias, particularly in the school sample where group testing was employed. Future studies may benefit from using a mixed-methods approach that incorporates qualitative interviews to capture adolescents’ subjective experiences and perspectives related to sex differences in suicidal ideation and its relationship to depression and NSSI. This could provide a more comprehensive understanding to illuminate the quantitative findings. Another limitation is the integration of clinical and school samples without controlling for potential differences. The clinical sample may have higher levels of psychopathology and different characteristics compared to the school sample, which could influence the observed relationships between SI, depression, NSSI, and related factors. Future research should more closely examine and control for sample characteristics to ensure that the findings are not confounded by differences between clinical and non-clinical populations.

## Conclusion

The sex differences in the associations between SI, depression and NSSI found in this study provide a useful clinical insight: we need to take full account of sex differences when using depression and NSSI to predict SI. Relatively little attention has been paid to these associations, and network analysis is a good tool for representing them. Although we have used emotional reactivity as a preliminary explanation for sex differences, the underlying mechanisms are not well understood. More questions should be explored, such as: are the sex differences in these associations unique to Chinese adolescents, or are they cross-cultural? What about adults? What is the mechanism behind this association?

### Electronic supplementary material

**Additional file 1: Fig. S1.** Two estimated network structures based on nonclinical (left) and clinical (right) samples. **Fig. S2.** Centrality indices for the nodes in the nonclinical and clinical networks, including those for strength, betweenness, closeness, and expected influence. Red and blue represent female and male networks, respectively. **Fig. S3.** Forest plot of the odds ratios in the logistic regression. The red numbers indicate ORs less than 1 and the blue numbers indicate ORs equal to or greater than 1. Note that the OR value is large for NSSI because it’s a categorical variable. **Table S1.** Logistic Regression Performed to Ascertain the Effects of Various Factors on the Likelihood of SI.

## Data Availability

Research data supporting the findings of this study are available upon reasonable request. The data that support the findings of this study are available on request from the corresponding author, Wen-Jing Yan.
