# Supplementary material for: Adolescent suicidal ideation: dissecting the role of sex in depression and NSSI predictors
Source: Child Adolesc Psychiatry Ment Health. 2024 Jun 6;18:68. doi: 10.1186/s13034-024-00741-z (PMC11157781; doi:10.1186/s13034-024-00741-z)
Supplement: Supplementary file 1 — Additional file 1: Fig. S1. Two estimated network structures based on nonclinical (left) and clinical (right) samples. Fig. S2. Centrality indices for the nodes in the nonclinical and clinical networks, including those for strength, betweenness, closeness, and expected influence. Red and blue represent female and male networks, respectively. Fig. S3. Forest plot of the odds ratios in the logistic regression. The red numbers indicate ORs less than 1 and the blue numbers indicate ORs equal to or greater than 1. Note that the OR value is large for NSSI because it’s a categorical variable. Table S1. Logistic Regression Performed to Ascertain the Effects of Various Factors on the Likelihood of SI. [file 13034_2024_741_MOESM1_ESM.docx]

Additional file 1: Fig. S1 Two estimated network structures based on nonclinical (left) and clinical (right) samples. The networks show the relationships among variables, including symptoms (SI, NSSI, depression, and anxiety) and psychological characteristics (ERS, SPSI, and DERS). The edge weights are the regression coefficients with regularization. Blue edges represent positive relationships and red edges indicate negative relationships. The thickness of the edge reflects the magnitude of the relationship.

Additional file 1: Fig. S2. Centrality indices for the nodes in the nonclinical and clinical networks, including those for strength, betweenness, closeness, and expected influence. Red and blue represent female and male networks, respectively.

Additional file 1: Table S1 Logistic Regression Performed to Ascertain the Effects of Various Factors on the Likelihood of SI

|  | Nonclinical | | | | Clinical | | | |
| --- | --- | --- | --- | --- | --- | --- | --- | --- |
|  | *B* | *SE* | *p* | *OR* | *B* | *SE* | *p* | *OR* |
| Sensitivity | .088 | .059 | .135 | 1.092 | -.122 | .060 | .042 | .885 |
| Intensity | -.115 | .078 | .140 | .891 | .093 | .075 | .215 | 1.097 |
| Persistency | .308 | .122 | .012 | 1.360 | .006 | .110 | .959 | 1.006 |
| Lack of Awareness | -.049 | .063 | .440 | .952 | .199 | .063 | .002 | 1.220 |
| Clarity | .084 | .091 | .357 | 1.088 | -.113 | .084 | .179 | .894 |
| Nonacceptance | -.090 | .062 | .146 | .914 | .000 | .062 | .994 | 1.000 |
| Impulses | -.007 | .056 | .898 | .993 | .010 | .055 | .849 | 1.010 |
| Goals | -.038 | .065 | .557 | .963 | .014 | .075 | .849 | 1.014 |
| Strategies | -.053 | .056 | .342 | .948 | .106 | .060 | .078 | 1.112 |
| NSSI | 1.586 | .460 | .001 | 4.884 | 1.838 | .506 | .000 | 6.284 |
| RPS | -.054 | .037 | .141 | .947 | .021 | .033 | .516 | 1.022 |
| AS | -.047 | .053 | .379 | .954 | .098 | .059 | .098 | 1.104 |
| ICS | .034 | .079 | .670 | 1.034 | -.002 | .089 | .978 | .998 |
| PPO | .015 | .095 | .878 | 1.015 | -.021 | .114 | .853 | .979 |
| NPO | -.006 | .073 | .934 | .994 | -.016 | .077 | .836 | .984 |
| Anxiety | .070 | .084 | .410 | 1.072 | .262 | .109 | .017 | 1.300 |
| Depression | .178 | .078 | .022 | 1.195 | -.007 | .080 | .930 | .993 |


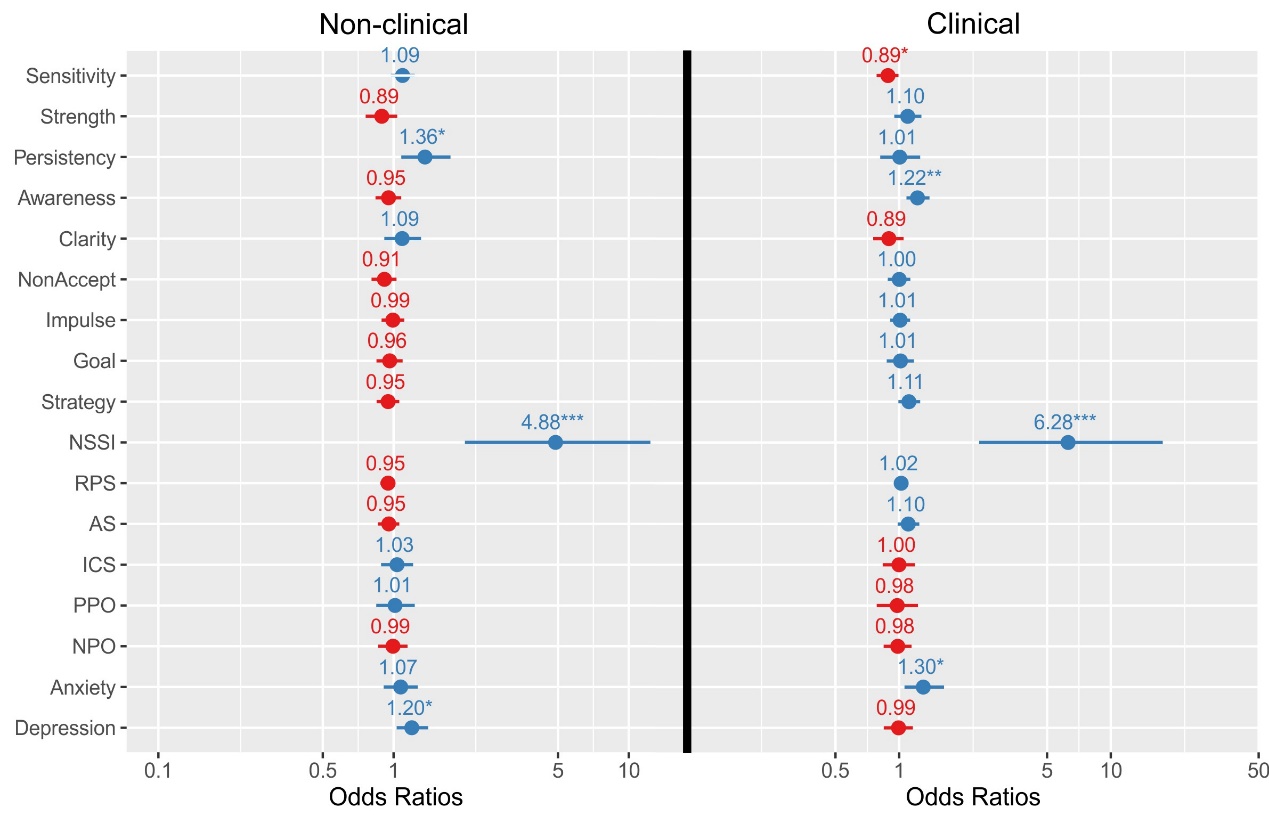


Additional file 1: Fig. S3 Forest plot of the odds ratios in the logistic regression. The red numbers indicate ORs less than 1 and the blue numbers indicate ORs equal to or greater than 1. Note that the OR value is large for NSSI because it’s a categorical variable.
